# Key predictors of fertility: Exploring the role of Vitamin-D

**DOI:** 10.12669/pjms.40.10.9774

**Published:** 2024-11

**Authors:** Huma Salahuddin, Samar Zaki, Mussarat Ashraf, Rehana Rehman

**Affiliations:** 1 Huma Salahuddin, Department of Physiology, Ziauddin Medical College, Karachi, Pakistan.; 2 Samar Zaki, Assistant Professor, Family Medicine, Aga Khan University, Karachi, Pakistan; 3 Mussarat Ashraf, Research Specialist, Department of Biological and Biomedical Sciences, Aga Khan University, Karachi, Pakistan; 4 Rehana Rehman, Professor, Department of Biological and Biomedical Sciences, Aga Khan University, Karachi, Pakistan

**Keywords:** Vitamin-D, Follicle Stimulating Hormone, Luteinizing Hormone, Anti Mullerian Hormone, Infertility, Reproductive Health

## Abstract

**Background & Objectives:**

The relationship of Vitamin-D (VD) with Luteinizing Hormone (LH), Follicle stimulating Hormone (FSH) and Anti Mullerian Hormone (AMH) is recognized. This study was conducted to determine whether VD is a predictor of fertility and investigate its relationship with female reproductive hormones and markers of ovarian reserve in female population of Karachi.

**Methods:**

A cross-sectional study was performed from July 2020 to June 2022 at the Aga Khan University Hospital (AKUH) with recruitment of 135 fertile and infertile subjects. VD, AMH, FSH and LH levels were estimated by ‘Enzyme Linked Immunosorbent assay (ELISA)’. By applying binary logistic regression, variables with p-value < 0.25 in univariate analysis were used for multivariate regression model and adjusted odds ratios were computed. In multivariate analysis, significance was p-value ≤ 0.05.

**Results:**

VD deficiency was observed in all infertile female subjects. In univariate regression analysis, FSH, LH, LH/FSH ratio and VD levels turned out to be significant. The Multivariate analysis represented that for every one ng/mL increase in VD level, the odds of fertility were expected to be 50.154 times higher with p-value < 0.001. The positive correlation of VD with FSH was significant.

**Conclusion:**

VD deficiency was prevalent in all infertile female subjects. VD levels were strong predictor of fertility in the study population. The significant association of VD with FSH explained the indirect effect of VD on follicular development and ovarian functions however no relationship of VD with ovarian reserve was exhibited.

## INTRODUCTION

Infertility can be attributed to numerous factors, ranging from hormonal imbalances and structural issues to genetic and epigenetic conditions.^1^ In Pakistan, prevalence of infertility is estimated to be 22% with a number of challenges faced by the infertile couples.^2^,^3^

The interaction of Vitamin-D (VD) and its impact on reproductive health has emerged as a particularly intriguing area of research. The vitamin traditionally recognized for its indispensable role in calcium metabolism and bone health has influence on various other physiological systems.^4^ VD role in development of follicles and synthesis of hormones by ovaries is supported by the presence of VD receptors in the ovaries.^5^ Anti-Müllerian hormone (AMH) is a marker for ovarian reserve; effect of VD on AMH can be justified by the existence of the ‘VDRE’ order in the gene promoter region of AMH.^5^,^6^ Optimal levels of VD, LH,FSH and AMH contribute in female reproductive health.^7^,^8^. VD increases the production of progesterone, estrogen and estradiol, and causes triggering of FSH receptor genes.^9^ Furthermore, VD supplementation has been used to improve the clinical pregnancy outcomes of infertile females in randomized and cohort studies.^10^

Considering the function of VD in health and fertility we came up with the following research questions:

- Is Vitamin-D a predictor of acquiring fertility in female population of Pakistan?
- Is there an association of VD with hormones of reproduction and marker of ovarian reserve?
- This study was conducted to determine whether VD is a predictor of fertility and investigate its relationship with female reproductive hormones and markers of ovarian reserve in a selected female population of Karachi.

## METHODS

It was a cross-sectional study, carried out from July 2020 to June 2022 at the AKUH with collaboration of Australian Concept of Infertility Medical Centre (ACIMC), Karachi, Pakistan.

### Ethical Approval:

The study was approved from ‘Ethical Review Committee (ERC: 2020-0314-14433, dated: September 14, 2021), Aga Khan University, Karachi, Pakistan’.

The sample size was calculated by using Open-Source Epidemiologic Statistics for Public Health keeping Pakistan’s infertility rate of 23% (2) and to accomplish 80% power and detecting an odds ratio of at minimum two.


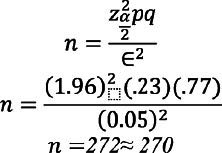


A total 288 females were recruited (140 fertile and 148 infertile females), out of which 135 infertile and 135 fertile females completed the study. Written informed consent was acquired from all study participants, general health checkup, measurement of height and weight and body mass index (BMI) calculation was done.

### Inclusion Criteria:

Females in the age range of 20–45 years bearing a child less than two years of age from all ethnic backgrounds were recruited as control subjects. The cases included female subjects between the age range of 20-45 years, from all ethnic backgrounds. All causes of infertility for more than two years duration was included as cases.

### Exclusion Criteria:

Females taking contraceptive pills during the last three months and suffering from diabetes mellitus, tuberculosis, thyroid problems and other endocrine disorders were excluded from the study.

Blood sample (5 ml) was collected in a tube, serum was separated after centrifugation and stored for further investigation. Serum levels of FSH and LH were determined by commercially available kits; ‘Human FSH Enzyme Immunoassay Kit (Cat. No. DKO010, Diametra Immuno Assays S.A. Belgium’; analytical sensitivity 0.17 mIU/mL and detection range was 0-100 mIU/mL) and ‘Human LH Enzyme Immunoassay Kit (Cat. No DKO009, Diametra source Immuno Assays S.A. Belgium’, detection range 5.0- 200 mIU/mL) respectively. Serum Vitamin-D was estimated with commercially available ‘25(OH) Vitamin-D Enzyme linked immunosorbent assay kit (Cat. No. ab213966, Abcam, Waltham, MA 02453, USA’, analytical sensitivity 1.98 ng/ml, detection range of 0.5- 1010 ng/ml) according to manufacturer’s protocol. Stratification of Vitamin-D levels into deficient (levels<20 ng/ml), insufficient (between 21 to 29.9 ng/ml) and sufficient (values>30 ng/ml) were considered 11 to compare Vitamin-D status in fertile and infertile females. Human AMH was analyzed by ‘AMH Enzyme linked immunosorbent assay kit (Cat# E1052Hu, Bioassay Technology Laboratory)’. The analytical sensitivity of the kit was 0.01 ng/ml with a detection range of 0.05- 15 ng/ml.

### Statistical Analysis:

The data was analyzed by using SPSS Version 24. The normality of quantitative variables i.e. age at marriage, duration of marriage, weight, height, BMI, FSH, LH, Vitamin-D level, AMH and LH/FSH ratio was assessed by using Shapiro-Wilk test. Normally distributed data was represented by Mean±SD while skewed data was represented by Med^IQR^ for fertile and infertile groups. The data of BMI groups, Vitamin-D, type of infertility and cause of infertility was expressed as frequency and percentages. Spearman’s rho correlation was applied to assess the correlation of Vitamin-D with LH, FSH and LH/FSH ratio. Binary logistic regression was applied to assess the relationship of multiple predictors with fertility and to control the confounding variables by univariate and multivariate analysis. From univariate analysis, the variables with P-value < 0.25 and other important variables irrespective of significance level, were used to build multivariate regression model to compute adjusted odds ratios. For multivariate analysis, the p-value ≤ 0.05 was considered statistically significant.

## RESULTS

Females (270) 135 fertile and 135 infertile completed this study. Among the 135 infertile women in the study, primary infertility accounted for 100 cases (74%), whereas secondary infertility comprised of 35 cases (26%). Polycystic ovary syndrome (PCOS) was the most common cause of infertility, (43.7%) participants, followed by male factor (8.1%), endometriosis (10.4%), tubal blockage (8.1%), and unexplained factors (29.6%). Vitamin-D deficiency was seen in all 135 (62.8%) infertile females and in 80 (37.2%) fertile females, p value <0.001. The demographic and clinical characteristics of both groups are compared in Table-1, which shows that height, FSH and Vitamin-D levels are statistically significantly different in fertile and infertile females with p-values 0.017, 0.009 and < 0.001 respectively.

**Table-I:** Comparison of Demographic and Clinical characteristics between Fertile and Infertile Females (n = 270)

Variables	Fertile females (n=135)	Infertile females (n=135)	P-value

	Med [IQR]	Med [IQR]	
Age (Years)	31 [9]	30 [10]	0.864
Age at marriage (Years)	22 [7]	22 [8]	0.286
Duration of marriage (Years)	7 [5]	7 [9]	0.773
Weight (kg)	70 [14]	71 [21]	0.947
Height (cm)	167 [4]	166 [3]	0.017*
BMI (kg/m^2^)	25.059 [4.95]	25.5593 [6.24]	0.424
FSH (mIU/mL)	6.4 [1.1]	6 [2.1]	0.009*
LH (IU/mL)	6.22 [1.97]	6 [4.17]	0.777
Vitamin-D level (ng/mL)	18.916 [6.05]	16.916 [3.53]	< 0.001*
AMH (ng/mL)	2.4 [4.21]	2.4 [3.4]	0.724
LH/FSH ratio	0.9194 [0.35]	1 [1.02]	0.614

Mann-Whitney U-test applied,

* represents significant p-value.

In Table-II the univariate analysis indicates that for every one-year increase in age, the odds of fertility were expected to be 0.8% lower (P-value > 0.25). For every one mIU/mL increase in FSH, the odds of fertility were expected to be 2.9% lower (P-value < 0.25). For every one IU/mL increase in LH, the odds of fertility were expected to be 6.9% lower (P-value < 0.25). For every one ng/mL increase in AMH, the odds of fertility were expected to be 3.3% higher (P-value > 0.25). For every 1 kg/m2 increase in BMI, the odds of fertility are expected to be 2.9% lower. Since P-value > 0.25, so statistically there is an insignificant association between BMI and fertility. For every one ng/mL increase in Vitamin-D level, the odds of fertility were expected to be 29.194 times higher. Since the P-value was < 0.25, so statistically there was a significant association between Vitamin-D level and fertility. Hence, we included VD level in multivariate regression model. The Multivariate analysis results represented the statistically significant association of Vitamin-D levels with fertility. For every 1 ng/mL increase in VD level, the odds of fertility were expected to be 50.154 times higher with p-value < 0.001.

**Table-II:** Binary Logistic Regression to determine predictors of fertility.

Independent variables	Univariate Analysis		Multivariate Analysis	

	Unadjusted OR (95% CI)	P-value	Adjusted OR (95% CI)	P-value
Age (Years)	0.992 (0.952 – 1.035)	0.718	1.043 (0.718 – 1.516)	0.824
Follicle Stimulating Hormone (mIU/mL)	0.971 (0.933 – 1.010)	0.140*	0.661 (0.368 – 1.188)	0.167
Luteinizing Hormone (IU/mL)	0.931 (0.872 – 0.994)	0.033*	1.705 (0.792 – 3.671)	0.173
Anti-Mullerian Hormone (ng/mL)	1.033 (0.967 – 1.104)	0.331	1.322 (0.990 – 1.765)	0.059
Body Mass Index (BMI kg/m^2^)	0.971 (0.910 – 1.035)	0.366	1.082 (0.878 – 1.332)	0.459
Vitamin-D levels (ng/mL)	29.194 (11.101 – 76.773)	< 0.001*	50.154 (12.950 – 194.233)	< 0.001**

* Significant at P-value < 0.25 for Univariate analysis,xs

** Significant at P-value < 0.05 for Multivariate analysis, the variables used for adjustment are age, FSH, LH, AMH, BMI and Vitamin-D levels.

Table-III represents that VD levels had a weak insignificant negative correlation with LH, and LH/FSH ratio but positively correlated with FSH (p-value = 0.051). In the infertile females group, VD levels are positively correlated with LH and LH/FSH ratio having a negligible correlation (r = 0.023, p-value = 0.795 and r = 0.012, p-value = 0.889 respectively), while negatively correlated with FSH having a negligible correlation (r = -0.060, p-value = 0.487). It also shows that in fertile females, the VD levels are negatively weekly correlated with LH and LH/FSH ratio (r = -0.213, p-value = 0.013 and r = -0.174, p-value = 0.043 respectively), while positively correlated with FSH having a negligible correlation (r = 0.018, p-value = 0.833).

**Table-III:** Relationship of Vitamin-D with LH, FSH and LH/FSH ratio.

Study Population (n=270)			

Variables	Med ^IQR^	Correlation (r)	P-value
Vitamin-D levels	16.916 ^2.76^	- 0.026	0.675
LH	6 ^2.75^		
Vitamin-D levels	16.916 ^2.76^	0.119	0.051
FSH	6.4 ^1.5^		
Vitamin-D levels	16.916 ^2.76^	- 0.062	0.307
LH/FSH ratio	0.9366 ^0.52^

***Infertile Group (n=135)***			

Vitamin-D levels	16.916 [3.53]	0.023	0.795
LH	6 [4.17]		
Vitamin-D levels	16.916 [3.53]	-0.060	0.487
FSH	6 [2.1]		
Vitamin-D levels	16.916 [3.53]	0.012	0.889
LH/FSH ratio	1 [1.02]

***Fertile Group (n=135)***			

Vitamin-D levels	18.916 [6.05]	-0.213	0.013
LH	6.22 [1.97]		
Vitamin-D levels	18.916 [6.05]	0.018	0.833
FSH	6.4 [1.1]		
Vitamin-D levels	18.916 [6.05]	-0.174	0.043
LH/FSH ratio	0.9194 [0.35]		

*Spearman’s rho correlation applied.

## DISCUSSION

VD plays an important role in reproductive functions of the body. The exact mechanism by which VD influences reproductive physiology is not known, however, several studies have explored the association of VD with different markers of fertility^12^. In our study, VD deficiency was observed in all infertile female subjects. The impact of VD deficiency on infertility is supported by existing literature in many population-based studies.^13^,^14^

A meta-analysis of interventional studies has documented that VD supplementation appreciably improves AMH levels particularly in females with normal or diminished ovarian reserve.^15^ Our study however, did not demonstrate any association of VD with AMH. Currently, whether VD may influence ovarian reserve has been a controversial issue. Studies have demonstrated a positive correlation between VD levels and ovarian reserve markers^15^, while other studies reported negative findings^12^.A recent cross-sectional study showed no significant association between serum VD and AMH levels in women with infertility^16^.This finding of lack of association between VD and AMH is also supported by a retrospective cohort study in which no cause effect relationship of VD with ovarian reserve parameters (AMH and FSH) was observed.^17^ The results of a systematic review including 18 observational and 6 interventional studies have shown that the relationship between VD and AMH is complex, and recommended that large randomized trials of VD supplementation are necessary to gain more insight into potential benefit of VD to female fertility^18^. Szafarowska et al. recently reported that polymorphisms in the VD receptor (VDR) gene are associated with elevated AMH levels in polycystic ovarian syndrome (PCOS). This finding supports the possible influence of VD on AMH levels in PCOS patients. Such genetic variations may contribute to differences in the findings on the association between VD and AMH levels among different studies^19^.

High FSH and LH were associated with decreased fertility odds. These findings are consistent with previous research, which also highlighted the importance of hormonal balance in female fertility.^20^-^22^ Our study showed a negative correlation of VD with FSH which is supported by literature^23^

Our study indicated that infertile females tend to have greater BMI in comparison to fertile females; results concur with previous studies.^24^ Although BMI was not a predictor of infertility (insignificant p value) yet the importance of weight management and its impact on reproductive health cannot be overruled.

### Limitations:

Our study was unicentric, had limited sample size and there was non-normal distribution of some variables. The study though considered multiple variables, like FSH, LH, LH/FSH ratio and BMI, yet a number of factors could not be considered as predictors of infertility. The data used to generate the study findings was cross sectional, however a longitudinal study should be used to examine the relationship of serum AMH with VD in individuals over the four seasons. However, these findings have the potential to enhance patient-centered care, guide tailored interventions, inform public health initiatives, and devise public strategies and policies ultimately contributing to improved fertility outcomes and reproductive health.25

## CONCLUSION

A comprehensive relationship between VD deficiency, FSH levels, and infertility was explored in the female study population, however no relationship with AMH was exhibited. VD deficiency was present in all infertile female subjects. The levels of VD were strong predictor of fertility in the study population. A significant association of VD with FSH relays cause effect explanation of the indirect effect of VD on follicular development and ovarian functions.

### Authors’ Contribution:

**RR:** Designed and supervised the study. **HA** and **SZ:** Executed the whole project from data collection till data analysis. **MA** performed the benchwork and took part in the analysis and write-up of the manuscript. All authors took part in the write-up of manuscript, read and revised the content, take responsibility for the integrity of the study.
